# Emerging Roles of Energy Metabolism in Ferroptosis Regulation of Tumor Cells

**DOI:** 10.1002/advs.202100997

**Published:** 2021-10-10

**Authors:** Xuemei Yao, Wei Li, De Fang, Chuyu Xiao, Xiao Wu, Menghuan Li, Zhong Luo

**Affiliations:** ^1^ School of Life Science Chongqing University Chongqing 400044 China; ^2^ Breast Cancer Center Chongqing Key Laboratory of Translational Research for Cancer Metastasis and Individualized Treatment Chongqing University Cancer Hospital Chongqing 400044 P. R. China

**Keywords:** cellular energy metabolism, ferroptosis, glucose, glutamine

## Abstract

Ferroptosis is a new form of regulated cell death, which is characterized by the iron‐dependent accumulation of lethal lipid peroxides and involved in many critical diseases. Recent reports revealed that cellular energy metabolism activities such as glycolysis, pentose phosphate pathway (PPP), and tricarboxylic acid cycle are involved in the regulation of key ferroptosis markers such as reduced nicotinamide adenine dinucleotide phosphate (NADPH), glutathione (GSH), and reactive oxygen species (ROS), therefore imposing potential regulatory roles in ferroptosis. Remarkably, tumor cells can activate adaptive metabolic responses to inhibit ferroptosis for self‐preservation such as the upregulation of glycolysis and PPP. Due to the rapid proliferation of tumor cells and the intensified metabolic rate, tumor energy metabolism has become a target for disrupting the redox homeostasis and induce ferroptosis. Based on these emerging insights, regulatory impact of those‐tumor specific metabolic aberrations is systematically characterized, such as rewired glucose metabolism and metabolic compensation through glutamine utilization on ferroptosis and analyzed the underlying molecular mechanisms. Additionally, those ferroptosis‐based therapeutic strategies are also discussed by exploiting those metabolic vulnerabilities, which may open up new avenues for tumor treatment in a clinical context.

## Graphical abstract

Ferroptosis is regulated by a variety of metabolic pathways, but the involvement of energy metabolism is rarely investigated. This review discusses the functional roles of various tumor metabolic phenotypes on ferroptosis regulation in the tumor landscape, which may improve the understanding of the tumor ferroptosis network and provide implications for the development of novel ferroptosis‐based therapies.

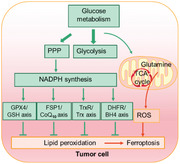

## 1 Introduction

**Figure 1:**
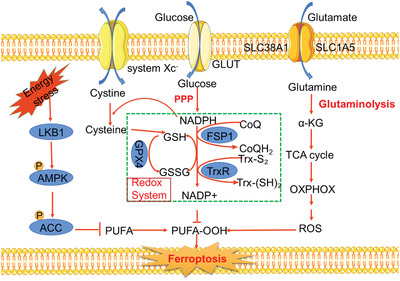
Schematic illustration showing the involvement of cellular energy metabolism in the ferroptosis regulatory network.

Despite the tremendous advance in tumor diagnosis and therapy in recent decades, it is still one of the greatest threats to human health and the major causes of death each year, which has become a severe burden to both the patients themselves and the whole society.^[^ ^1^, ^2^, ^3^, ^4^ ^]^ Currently, the mainstream tumor chemotherapies employ apoptosis‐inducing modalities to kill or inhibit tumor cells.^[^ ^5^ ^]^ However, it has become increasingly evident that tumor cells could demonstrate intrinsic or acquired resistance to these apoptosis‐dependent antitumor modalities and substantially increase the risk of treatment failure and post‐treatment relapse.^[^ ^6^, ^7^, ^8^, ^9^ ^]^ Therefore, it is of both clinical and practical significance to discover new non‐apoptotic regulated cell death pathways and explore their therapeutic usage.^[^ ^10^ ^]^ In 2003, Stockwell et al. employed high‐throughput screening method to interrogate the cytotoxicity of around 23 550 synthetic lethal small‐molecule agents against engineered tumorigenic cells and firstly observed that the two compounds of Erastin and RSL3 could selectively kill tumor cells expressing Small T oncoprotein and oncogenic RAS, of which the morphological features were distinctively different from those extensively studied cell death pathways such as apoptosis or necrosis.^[^ ^11^, ^12^ ^]^ In a follow‐up study by the same group in 2012, the authors thoroughly investigated the morphological, biochemical, and genetic features of this emerging route of regulated cell death and evidently confirmed that it was different from any known form of cell death routes such as apoptosis, necrosis or autophagy.^[^ ^13^ ^]^ Due to its dependence on cellular iron but not other metal ions, this novel form of regulated cell death was eventually termed “ferroptosis”.^[^ ^14^, ^15^ ^]^ The continuous progress in ferroptosis research has substantially expanded the arsenal of ferroptosis‐inducing agents for potential therapies, wherein some old drugs may also present pro‐ferroptosis activities. For instance, Sorafenib, originally used as a US Food&Drug Administration (FDA)‐approved inhibitor of multiple oncogenic kinases for tumor therapy, could induce ferroptosis by inhibiting the activity of SLC7A11 in a similar manner to erastin.^[^ ^16^, ^17^ ^]^ Meanwhile, the FDA‐approved anti‐inflammatory agent Salazosulfapyridine could also induce ferroptosis by suppressing the System Xc^–^‐mediated exchange of cystine and glutamate.^[^ ^18^ ^]^ From a morphological perspective, ferroptotic cells usually demonstrate mitochondrial volume shrinkage, increasing mitochondrial membrane density and decreasing mitochondrial cristae, while no obvious changes were observed in the morphology of the cell nucleus and chromosomes.^[^ ^19^ ^]^ Meanwhile, ferroptotic cells are also associated with a series of characteristic biochemical features including the universal upregulation of cellular reactive oxygen species (ROS) and lipid ROS levels, accompanied with alterations in the expression levels of metabolism‐related genes such as glutathione peroxidase 4 (GPX4) and ferroptosis suppressor protein 1 (FSP1) (**Figure** **1**).^[^ ^20^, ^21^, ^22^, ^23^ ^]^ Interestingly, recent insights indicate that ferroptosis is intrinsically linked with multiple cellular metabolic pathways including energy, lipid, and amino acid metabolism, which directly affects the cell's susceptibility to lipid peroxidation and ferroptosis.^[^ ^24^, ^25^, ^26^, ^27^ ^]^

**Figure 2:**
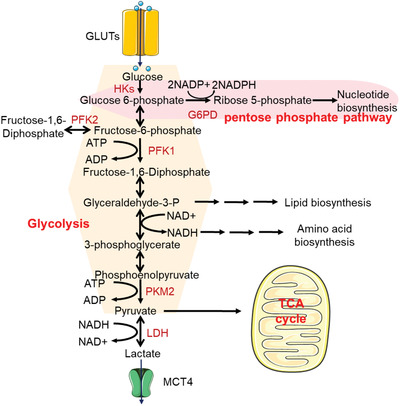
Schematic depiction of glucose metabolism in cellular environment.

In the pioneering study by Otto Warburg in 1920, the author first reported the discovery that tumor cells usually had much higher glycolysis rate than normal cells.^[^ ^28^ ^]^ Due to the central role of metabolic aberrations in the genesis and progression of various tumors, they are often evaluated as metabolic diseases.^[^ ^29^, ^30^, ^31^ ^]^ In the concept proposed by Otto Warburg, it was theorized that tumors were caused by certain pathological changes in mitochondria that disabled their ability of aerobic respiration.^[^ ^32^, ^33^ ^]^ To ensure self‐survival as well as to meet the increasing demand of biosynthetic precursors, tumor cells would activate other energy production pathways and rewire their glucose metabolism to aerobic glycolysis.^[^ ^34^, ^35^ ^]^ Remarkably, these mitochondrion‐disabled tumor cells would take in large amount of glucose and thus facilitate higher rate of glycolysis, which played critical roles in maintaining the adenosine triphosphate (ATP) and NADPH supply to support vital biological events such as macromolecular biosynthesis, membrane‐protein integration, maintenance of cross‐membrane ion gradients and DNA replication.^[^ ^36^ ^]^ Nonetheless, many of these energy metabolic aberrations converge on ferroptosis and profoundly affect its initiation and execution.^[^ ^37^ ^]^ Typically, glucose is the primary energy source and biosynthetic substrate for most cell types. Under normal conditions, glucose was first converted to pyruvate via the glycolysis route then entered the tricarboxylic acid cycle (TCA) cycle and oxidative phosphorylation (OXPHOS) processes for complete metabolization.^[^ ^38^, ^39^ ^]^ Alternatively, under hypoxic or malignant conditions, the glycolysis‐generated pyruvate was converted to lactate under the catalyzation of lactate dehydrogenase.^[^ ^40^ ^]^ Specifically, the internalized glucose molecules were first phosphorylated by hexokinase (HKs) to produce glucose 6‐phosphate, which would be further converted into converted to fructose‐6‐phosphate by glucose‐6‐phosphate isomerase to join the subsequent metabolic phases for energy production.^[^ ^41^ ^]^ Meanwhile, the fructose‐6‐phosphate may also be bypassed into the pentose phosphate pathway (PPP) by 6‐phosphate glucose dehydrogenase for complete oxidation, leading to the production of those necessary biosynthetic precursors and NADPH (**Figure** **2**).^[^ ^41^, ^42^ ^]^ Importantly, NADPH is not only an essential bioreductor for the biosynthesis of vital biomolecules including lipids, nucleic acids, and amino acids, but is also involved in the recycling of the oxidized GSH by glutathione reductase for maintaining the cellular redox homeostasis.^[^ ^43^, ^44^ ^]^ Based on the insights above, it could be concluded that the energy metabolism in tumor cells is one of the major regulatory forces of the ROS stress and antioxidant defense in the tumor cellular environment, indicating the strong correlation with ferroptosis. Nevertheless, there's still no systemic review on their relationship. In this review, we will provide a comprehensive summary on regulatory function of different energy metabolic routes on ferroptosis in tumor cells and discuss those metabolic vulnerabilities for the development of potential ferroptosis‐based tumor therapies.^[^ ^45^ ^]^

## 2 Glucose‐Dependent Energy Metabolism and Ferroptosis

**Figure 3:**
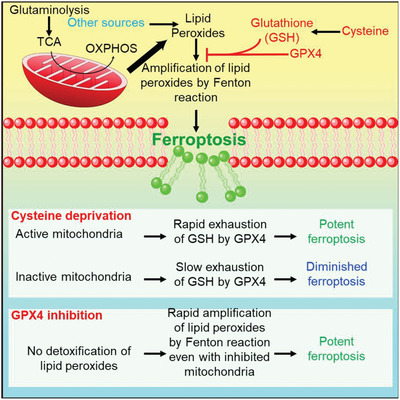
Role of mitochondria in the ferroptosis process. Reproduced with permission.^[^ ^19^ ^]^ Copyright 2018, Elsevier.

Mitochondrion is the primary site for oxidative respiration inside cells and also the major source of ROS.^[^ ^46^, ^47^ ^]^ It is well established that the glucose‐dependent mitochondrial bioenergetic processes are also accompanied by the transfer of electrons at Complex I and Complex III in the electron transport chain to molecular oxygen, leading to the generation of ROS during these catabolic reactions.^[^ ^48^, ^49^, ^50^ ^]^ Typical ROS produced through the mitochondrial respiration chain include H_2_O_2_, O_2_^−^, HO·, etc., and their biological functions are manifold. On one hand, ROS could act as signaling molecules to stimulate cell proliferation.^[^ ^51^, ^52^, ^53^ ^]^ On the other hand, the abnormal accumulation of ROS may also cause cellular damage and compromise cell survival.^[^ ^54^ ^]^ Further evidence was provided in the report by Jiang et al., in which the authors demonstrated that the TCA cycle and OXPHOS process have important roles in promoting ferroptosis induced by cysteine deprivation.^[^ ^19^, ^55^ ^]^ Remarkably, the authors showed that inhibiting the TCA cycle could suppress the mitochondrial membrane depolarization in tumor cells and interrupt the mitochondrial electron transport chain to reduce the cellular lipid ROS levels, thus inhibiting the ferroptosis of these tumor cells.^[^ ^19^ ^]^ Similar anti‐ferroptosis effects could also be achieved by inhibiting the activity of fumarate hydratase, which is an important metabolic enzyme involved in the TCA cycle.^[^ ^56^ ^]^ In a follow‐up study by Jiang et al., the authors identified that alpha‐ketoglutarate, an intermediate product in the TCA cycle, as well as its downstream products such as succinic acid and fumaric acid could all enhance the ferroptosis induced by cysteine depletion (**Figure** **3**).^[^ ^19^ ^]^ Alternative to those metabolites in the mitochondrial respiration process, it was also reported that the mitochondrial voltage‐dependent anion channel (VDAC) was involved in the Erastin‐induced ferroptosis. VDAC is a class of porin ion channels located on the outer mitochondrial membrane, which is responsible for controlling the exchange of ions and metabolites between the cytosol and the mitochondria.^[^ ^57^, ^58^ ^]^ Interestingly, erastin could inhibit the complexation of tubulin with VDAC and maintain VDAC in an opened state, consequently enhancing the mitochondrial respiration and increasing ROS production to promote ferroptosis.^[^ ^59^, ^60^, ^61^ ^]^ In contrast, downregulating the expression level of VDAC2/3 or treating cells with the ROS scavenger of *N*‐acetyl‐l‐cysteine (NAC) could reduce the mitochondrial ROS production, thus rescuing the cells from the erastin‐induced ferroptosis.^[^ ^60^ ^]^ The evidence above collectively demonstrates the indispensable regulatory role of mitochondrial energy production in the ferroptosis process.

It is well established that ROS produced during TCA cycle and OXPHOS could also act as secondary messengers to regulate various cellular activities.^[^ ^62^, ^63^ ^]^ However, the metabolic rates of tumor cells are usually accelerated to support their rapid and uncontrolled proliferation, which would lead to enhanced cellular ROS stress and render them intrinsically susceptible to ferroptosis. Consequently, the glucose metabolism in malignant tumor cells is usually rewired to glycolysis to meet their increasing demands for energy and biosynthetic precursors as well as maintenance of redox homeostasis to prevent ferroptosis. For instance, it has been observed that tumor cells have high glycolytic rate to compensate its intrinsically low ATP‐generating efficiency.^[^ ^64^, ^65^ ^]^ Meanwhile, the mitochondrial OXPHOS activity in these glycolytic tumor cells have been evidently inhibited to relieve the ROS stress. Nevertheless, the glycolysis‐induced suppression of OXPHOS is not a permanent state and could be reversed when the glycolysis becomes inhibited, accompanied with increasing susceptibility to mainstream chemotherapy as well as ferroptosis inducers.^[^ ^66^ ^]^ The underlying mechanism for the ferroptosis sensitization after glycolysis inhibition is that the metabolic rewiring to OXPHOS would elevate the cellular ROS levels to disrupt iron homeostasis and promote lipid peroxidation.^[^ ^19^ ^]^ Consistent with these insights, it has been observed that tumor cells undergoing erastin or RSL3‐induced ferroptosis showed substantially lower glycolytic activities, evidenced by the significant downregulation of three key glycolysis enzymes including HK II, platelet‐type phosphofructokinase and pyruvate kinase M2 (PKM2) in a recent report by Wang et al.^[^ ^67^, ^68^ ^]^ The insights above are immediate evidence that ferroptosis‐inducing mechanism for both erastin and RSL3 is by reversing the cellular metabolic pathway from glycolysis to OXPHOS, further indicating that the coordination between glycolysis and OXPHOS have profound impact on the cellular ROS stress and could regulate ferroptosis in a dynamic manner.

## 3 PPP Activity and Ferroptosis

**Figure 4:**
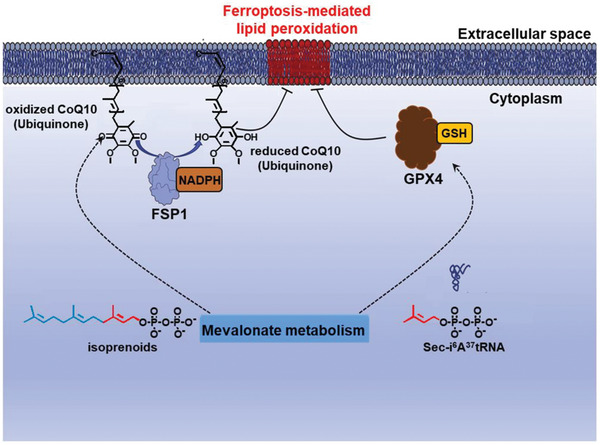
Schematic depiction of the FSP1‐mediated ferroptosis regulation, in which NADPH functions as a central bioreductor for the recycling of CoQ10. Reproduced with permission.^[^ ^22^ ^]^ Copyright 2019, Elsevier.

**Figure 5:**
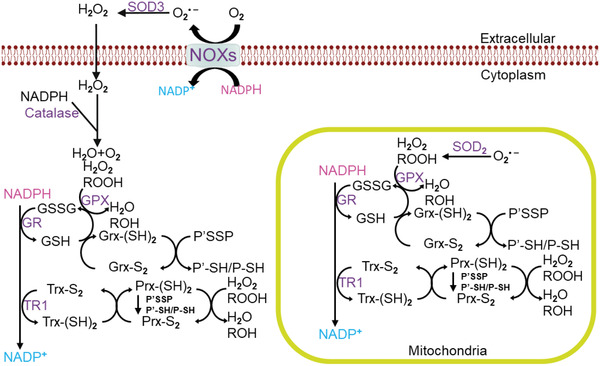
The regulatory roles of NADPH in maintaining the cellular redox homeostasis. Reproduced with permission.^[^ ^83^ ^]^ Copyright 2020, Chongqing Medical University. Production and hosting by Elsevier B.V.

Tumor cells not only exploit glucose as an energy source for the glycolysis‐mediated ATP production, but also show increasing activities of the PPP, through which glucose is consumed to obtain those biosynthetic precursors as well as to maintain the cellular redox homeostasis.^[^ ^69^ ^]^ Typically, PPP branches after the initial step of glycolysis, during which the intermediate metabolite glucose 6‐phosphate (G6P) was bypassed into the oxidative and non‐oxidative branches of PPP to synthesize ribose 5‐phosphate (precursor of nucleotide and nucleotide coenzyme),^[^ ^70^ ^]^ erythrose 4‐phosphate (precursor of aromatic amino acids) and NADPH.^[^ ^71^, ^72^, ^73^, ^74^ ^]^ Consistent with the central role of NADPH in the cellular metabolic network and redox homeostasis, it has been recently discovered that NADPH could regulate ferroptosis from multiple aspects. For instance, Wang et al. discovered that oxidoreductases on the endoplasmic reticulum such as NADPH‐cytochrome P450 reductase (POR) and NADH‐cytochrome b5 reductase (CYB5R1) were responsible for the catalyzation of lipid peroxidation during ferroptosis.^[^ ^75^ ^]^ Under normal conditions, POR and CYB5R1 would transfer the electrons of NADPH/NADH to the downstream proteins such as cytochromes P450.^[^ ^76^ ^]^ However, they may incidentally donate the electrons to the oxygen in the cellular environment under certain circumstances to generate H_2_O_2_, which would be further converted to hydroxyl radicals through the iron‐catalyzed Fenton reaction route. Due to the high reactivity of the hydroxyl radicals, they could cause the abstraction of hydrogen atoms from the methylene carbons in the polyunsaturated fatty acids (PUFAs) and readily initiate lipid peroxidation, leading to the disruption of the plasma membrane.^[^ ^77^, ^78^, ^79^ ^]^ NADPH could also act as electron carriers and donate electrons to enable the glutathione reductase‐mediated reduction from glutathione disulfide (GSSG) to glutathione (GSH).^[^ ^80^ ^]^ Meanwhile, NADPH could also support the SLC7A11‐mediated intake of cystine and further convert it into cysteine for GSH synthesis.^[^ ^81^, ^82^, ^83^, ^84^ ^]^ GSH would subsequently participate in the glutathione peroxidase 4 (GPX4)‐mediated elimination of lipid ROS to inhibit ferroptosis.^[^ ^23^, ^83^ ^]^ In addition to GSH regeneration and synthesis, NADPH also participates in the thioredoxin reductase (TR)‐mediated regeneration of thioredoxin (Trx) and form a functional system for the reduction of protein‐disulfides.^[^ ^83^, ^84^ ^]^ Specifically, the reduced Trx could be oxidized by donating a proton from the sulfhydryl group to reduce other protein‐disulfides, and the oxidized Trx then reacts with NADPH under the catalyzation of TR and is converted back to the reduced form for recycling.^[^ ^83^, ^84^, ^85^ ^]^ This system is a critical biochemical component in redox homeostasis, antiviral defense, and ferroptosis regulation. For instance, the report by Evijola Llabani et al. showed that a small‐molecule compound ferroptocide could inhibit Trx in a targeted manner and initiate ferroptotic cell death.^[^ ^86^ ^]^ There are also reports that NADPH is involved in a non‐GPX4‐dependent ferroptosis route by cooperating with ferroptosis suppressor protein 1 (FSP1) to reduce Coenzyme Q_10_ (CoQ10) to CoQ10‐H_2_, which is a potent antioxidant capable of preventing the propagation of lipid peroxidation on the plasma membrane (**Figures** **4** and **5**).^[^ ^20^, ^21^ ^]^ Mariluz Soula et al. demonstrated that NADPH could also affect the ferroptosis process by mediating the regeneration of BH4, which is an effective free radical trapping antioxidant capable of effectively clearing lipid peroxide to protect cells from ferroptotic death when GPX4 activity is inhibited. The underlying molecular mechanism is that NADPH could act as the cofactor to enable the regeneration of tetrahydrobiopterin (BH4) via difolate reductase (DHFR) in addition to the de novo guanosine triphosphate cyclohydrolase I (GCH1)‐mediated synthesis route.^[^ ^87^ ^]^ Extending from the evidence above, it was discovered that inhibiting the NAD+ kinase (NADK) could reduce the cellular NADPH levels and enhance their susceptibility to ferroptosis induction by typical ferroptosis inducers such as erastin, RSL3, and FIN56, further validating the potential application of NADPH as a biomarker for assessing ferroptosis sensitivity.^[^ ^88^, ^89^ ^]^ Recently, Leu et al. demonstrated that by replacing the proline (P47) at the codon 47 of the human p53 protein into serine (S47), the cells would show reduced glycolysis capacity while metabolism through PPP would be enhanced significantly, accompanied with increasing NADPH/NADP+ ratio.^[^ ^90^ ^]^ S47 cells showed distinctive metabolic profiled compared to P47 cells, characterized by lower ROS stress and expression of Activating Transcription Factor 4 (ATF4).^[^ ^90^ ^]^ However, enhancing the expression of ATF4 in S47 cells could reverse this trend and inhibit the PPP activities therein, leading to increasing sensitivity to ferroptosis‐inducing treatments.^[^ ^90^ ^]^ Overall, it could be concluded that the PPP‐generated NADPH could boost the antioxidant defense in tumor cells by supporting the biosynthesis of GSH, Trx, and CoQ10‐H_2_ to rescue them from ferroptosis, and targeting the abnormally elevated PPP in tumor cells could afford a robust strategy to disrupt the cellular redox homeostasis for ferroptosis‐based tumor therapy.

## 4 Energy Stress and Ferroptosis

**Figure 6:**
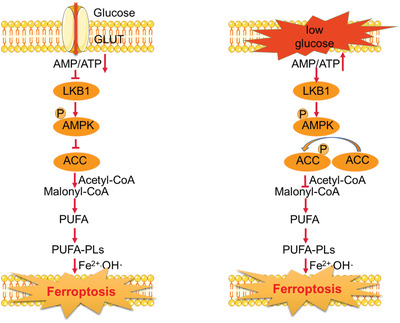
Impact of AMPK activation on ferroptosis under energy stress.

As already discussed above, glucose is one of the most important energy sources for tumor cells and glucose metabolism is fundamentally connected to the ferroptosis process. However, due to highly disorganized nature of blood vessels in rapidly proliferating tumors,^[^ ^91^ ^]^ some of the tumor cells may have limited blood supply and be forced into a glucose‐deprived state, thus suppressing those glucose‐metabolism routes such as glycolysis and PPP as well as severely compromising cellular energy supply and biosynthesis.^[^ ^92^ ^]^ To ensure cell survival, tumor cells would activate the energy crisis censor adenosine monophosphate ‐activated protein kinase (AMPK) to enhance ATP production and prevent its decomposition.^[^ ^93^, ^94^ ^]^ Specifically, the energy crisis due to glucose deprivation would elevate the AMP: ATP and adenosine diphosphate:ATP ratios and activate the phosphorylation of AMPK to phosphorylate the downstream effectors, thus promoting those ATP‐generating catabolic events while simultaneously inhibiting those ATP‐consuming anabolic events to maintain the ATP level.^[^ ^95^ ^]^ Recent studies indicate that AMPK would cause the phosphorylation of acetyl‐CoA carboxylase (ACC) to inhibit its activity and impose regulatory effect on tumor cell ferroptosis.^[^ ^37^, ^96^ ^]^ ACC is a rate‐limiting enzyme in the biosynthesis process of fatty acids, and it has been observed in lipidomic analysis that inhibiting ACC in tumor cells would cause a substantial decrease in the cellular abundance of ferroptosis‐susceptible PUFAs,^[^ ^97^ ^]^ thus decreasing their susceptibility to ferroptosis inducing treatments (**Figure** **6**). Extending from the role of AMPK in ferroptosis regulation, it has been reported that liver kinase B1 (LKB1), an upstream of AMPK, could also affect ferroptosis in the cellular environment.^[^ ^98^ ^]^ Typically, LKB1 is a master serine/threonine kinase and involved in numerous cellular events, especially for the metabolic response to tumor cells under energy stress.^[^ ^99^ ^]^ Gao et al. reported that LKB1 could enhance the phosphorylation of AMPK as well as the downstream ACC1 to inhibit the synthesis of PUFA.^[^ ^37^, ^55^ ^]^ Consistently, knocking down LKB1 would sensitize cells to the ferroptosis induced by erastin. Alternative to the impact on lipid synthesis, the energy stress‐induced AMPK activation could also compensate the PPP‐suppression‐induced reduction of cellular NADPH and enhance ferroptosis resistance. The underlying mechanism is that AMPK could phosphorylate and inhibit ACC1 and ACC2 to suppress the NADPH‐consuming synthesis of fatty acids, while at the same time enhancing the oxidization of fatty acids to replenish the NADPH supply.^[^ ^37^ ^]^ The discussions above evidently suggest that the AMPK activation under energy stress could simultaneously reduce the generation of ferroptosis‐susceptible fatty acids while maintaining the antioxidative defense, both of which would contribute to the ferroptosis resistance in tumor cells.

## 5 Glutamine‐Dependent Supplementation of TCA Cycle and Ferroptosis

**Figure 7:**
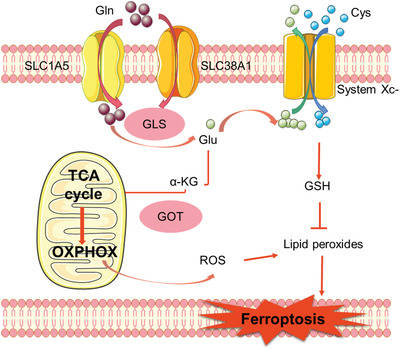
Schematic illustration on the role of glutamine metabolism in ferroptosis regulation. Gln: glutamine.

Glutamine is an important non‐essential amino acid in human body and widely involved in many metabolic events including the biosynthesis of proteins and GSH as well as energy production.^[^ ^100^, ^101^, ^102^ ^]^ Latest insights reveal although the mitochondrial respiration activities are disabled in tumor cells, those mitochondria are actually still functionally intact and the metabolic suppression is caused by the blockade of pyruvate flow to the mitochondria. Moreover, glutamine metabolism has regained increasing relevance on account of its biological roles and the crosstalk effect with glucose metabolism. Specifically, mitochondrial l‐glutamine catabolism is a critical process in tumor cells for maintaining mitochondrial potential and membrane integrity, and hyperglycolytic tumor cells tend to show greater dependence on l‐glutamine metabolism via CtBP‐SIRT4‐GDH axis,^[^ ^103^ ^]^ which is a commonly observed phenomenon in breast, ovarian and pancreatic cancers.^[^ ^104^, ^105^, ^106^ ^]^ Typically, glutamine is taken in by tumor cells primarily through SLC1A5 and SLC38A1 transporters and then converted into glutamate under the catalyzation of glutaminase (GLS).^[^ ^102^, ^107^ ^]^ It is important to note that GLS has two subtypes that are GLS1 and GLS2, and only GLS2 is involved in the ferroptosis process. The underlying molecular mechanism is that only glutamate generated by GLS2‐mediated glutaminolysis may contribute to *α*‐ketoglutarate (*α*‐KG) formation and subsequently enhance the production of oxidizable lipids to induce ferroptosis.^[^ ^56^, ^108^ ^]^ After entering the tumor mitochondria, the GLS2‐mediated glutamate could be converted to *α*‐KG under the catalyzation of aspartate aminotransferase (GOT) and glutamate dehydrogenase 1 (GLUD1) and thus maintain the TCA cycle function for metabolic compensation.^[^ ^108^ ^]^ It was also reported that tumor cells with intense TCA cycle activities were prone to glutamine addiction and demonstrating upregulated OXPHOS levels,^[^ ^62^ ^]^ thus elevating the cellular ROS stress and sensitizing tumor cells for ferroptosis (**Figure** **7**). Consistently, inhibiting the glutaminolysis process in tumor cells could suppress the TCA cycle activities therein and induce a series of functional changes in mitochondria such as reduced mitochondrial membrane potential depolarization and downregulation of mitochondrial electron transport chain activity, thus leading to reduced lipid ROS levels and enhanced ferroptosis resistance.^[^ ^19^, ^56^ ^]^ As shown in the report by Yang et al., treating tumor cells with a SLC1A5 inhibitor (miR‐137) or an aspartate aminotransferase (GOT1) inhibitor (miR‐9) could both suppress the glutaminolysis in melanoma cells by disrupting the expression and function of their corresponding targets and negatively affect ferroptosis, validating their therapeutic potential of the ferroptosis regulation mechanisms discussed above.^[^ ^109^, ^110^ ^]^

Recent insights also reveal that the glutamine metabolism in tumor cells is strongly affected by the cellular transport/metabolism of cystine.^[^ ^111^ ^]^ Specifically, System Xc‐ is a cystine/glutamate antiporter responsible for the regulation of cellular cysteine and glutamate levels, which is comprised of a light‐chain component (encoded by SLC7A11 gene) and a heavy‐chain component (encoded by SLC3A2 gene), in which the SLC7A11 subunit could take in extracellular cystine while simultaneously exporting intracellular glutamate at a 1:1 ratio.^[^ ^112^ ^]^ It should be noted that tumor cells are usually overexpressed with the SLC7A11 subunit to take in large amount of cystine and convert it into cysteine for the synthesis of GSH.^[^ ^113^, ^114^ ^]^ This is an important process in maintaining the redox homeostasis, but it may also cause the rapid loss of cellular glutamate. To sustain the uptake of extracellular cystine, tumor cells often have upregulated glutaminolysis activities to replenish the cellular glutamate supply, which underlies the dependence of SLC7A11‐overexpressed tumor cells on glutamine metabolism.^[^ ^115^, ^116^, ^117^ ^]^ Consequently, it has been theorized that cystine deprivation could also suppress the glutamate export and the glutaminolysis‐generated glutamate would be decomposed through TCA cycle, leading to the production of high level of ROS to promote ferroptosis. Based on this rationale, inhibiting key targets in tumor glutamine metabolism could offer new opportunities for ferroptosis‐based tumor therapy.

## 6 Therapeutic Implications of the Interaction between Energy Metabolism and Ferroptosis

Despite the tremendous progress in antitumor research in recent years, tumor therapy remains one of the great clinical challenges. Conventional treatment modalities such as chemotherapy and radiotherapy would inevitably induce adverse side effects against normal cells and tissues, and their efficacy would decline rapidly due to the emergence of intrinsic and acquired treatment resistance in tumor cells. Consequently, It is urgent to develop new treatment strategies based on alternative tumor inhibition mechanisms. Ferroptosis is a recently discovered route of regulated cell death, which is distinctively from those previously discovered form of regulated cell death such as apoptosis, pyroptosis, and necrosis in terms of morphological, biochemical, and genetic features and shows particular promise for therapeutic purposes.^[^ ^13^ ^]^ The primary driving force of ferroptosis is the cellular ROS stress generated by the energy metabolic activities. Excessive cellular ROS would react with the PUFA components in cell membrane under the catalysis of iron species to initiate and propagate lipid peroxidation, eventually leading to cell death.^[^ ^13^, ^118^ ^]^ Remarkably, the ROS levels in tumor cells are usually elevated compared to normal cells due to the intense metabolic activities, which renders them intrinsically susceptible to ferroptosis. These studies above collectively demonstrate the central role of energy metabolism in coordinating the tumor response to ferroptosis while also highlighting the potential application of ferroptosis as a therapeutic modality for tumor treatment.

As already discussed above, the cellular energy metabolism is directly connected to the ferroptosis process as it could regulate the antioxidative defense by mediating the synthesis of biomacromolecules and bioreductors such as NADPH.^[^ ^119^ ^]^ From a general perspective, tumor cells usually demonstrate upregulated glycolysis and PPP activities, which could not only inhibit the mitochondrial respiration to reduce ROS generation but also replenish the NADPH supply, thus contributing to the maintenance of the redox homeostasis to ensure cell survival. Meanwhile, several characteristic genetic mutations in tumor cells such as RAS, PI3K, and P53 could substantially boost the energy metabolism in tumor cells and affect the initiation and progression of ferroptosis therein.^[^ ^120^, ^121^, ^122^ ^]^ It could be thus concluded that disrupting the tumor energy metabolic pathways would not only alter the ferroptosis sensitivity of mutated tumor cells but also breading down their antioxidative defense to promote ferroptosis. Typically, Dannielle DeWaal et al. reported that downregulating the glycolysis in tumor cells could force the cellular glucose to enter the TCA cycles for OXPHOS, thus elevating the ferroptosis susceptibility of tumor cells under cystine deprivation.^[^ ^123^ ^]^ Meanwhile, interrupting the PPP route in tumor cells could downregulate the NADPH levels to inhibit the expression of downstream bioreductors such as GSH, CoQ10, and Trx, which was also beneficial for enhancing ferroptosis in the tumor landscape.^[^ ^20^ ^]^ Additionally, the intense glycolysis of tumor cells would also reshape the tumor microenvironment, leading to the upregulation of lactate levels in the tumor extracellular compartment.^[^ ^124^ ^]^ Interestingly, lactate could not only be exploited as an energy source by tumor cells, but also function as a signaling molecule to activate the hydroxycarboxylic acid receptor 1 (HCAR1) on tumor cell membrane to promote the production of anti‐ferroptotic monounsaturated fatty acid (MUFA).^[^ ^125^ ^]^ Based on this rationale, blocking the tumor lactate signaling pathways could be a viable strategy for ferroptosis sensitization. According to the report by Zhao et al., the authors confirmed that the lactate species in the tumor microenvironment could upregulate the production of MUFAs in tumor cells via the HCAR1/MCT1‐SREPB1‐SCD1 axis, which was capable of suppressing the lipid peroxidation in hepatocellular carcinoma (HCC) cells and ensuring the survival of HCC cells under ferroptosis induction with erastin or RSL3.^[^ ^125^ ^]^ In contrast, treating HCC cells with the combination of an MCT1 inhibitor (AZD3965) and RSL3 could efficiently initiate lipid peroxidation and induce ferroptosis both in vitro and in vivo. This study provides direct evidence on the anti‐ferroptotic roles of HCAR1 and MCT1 in tumor cells, highlighting their potential applications as molecular targets for ferroptosis‐based HCC therapy.^[^ ^125^ ^]^

It is well established that the ferroptosis process could be affected by several key factors including GSH synthesis, GPX4 expression, activity of ferroptosis‐related enzymes and cellular iron abundance, and preliminary research exploiting these vulnerabilities has already generated positive therapeutic responses on xenograft tumor models in vivo.^[^ ^126–15^ ^]^ However, James A. Olzmann et al. reported that certain types of tumor cells were still insensitive to ferroptosis induction after GPX4 inhibition or knockdown, indicating that ferroptosis is actually regulated by a complex network and more regulators still remain to be discovered. For instance, tumor cells were capable of removing the lipid ROS via FSP1–CoQ10–NAD(P)H pathway in a non‐GPX4‐dependent manner, which may act in parallel with the GSH‐dependent antioxidative defense to prevent ferroptosis.^[^ ^20^, ^21^ ^]^ In another study by Jiang et al., the authors discovered that immortalized MCF10A cells showed upregulated Prominin2 expression under pro‐ferroptotic stimuli, which is a pentaspanin protein capable of promoting the formation and secretion of ferritin‐containing multivesicular bodies and exosomes. Specifically, inhibiting GPX4 in breast cancer cells would activate the prominin2‐MVB‐exosome‐ferritin pathway to reduce the cellular iron abundance and prevent ferroptotic cell death.^[^ ^127^ ^]^ Recently, Xuejun Jiang et al. reported that PI3K‐AKT‐mTOR pathway mutation, a common mutation that frequently occurs in a wide variety of cancers, could enhance the ferroptosis resistance of cancer cells by upregulating the synthesis of anti‐ferroptotic fatty acids through the SREBP1‐SCD1 axis.^[^ ^120^ ^]^ The authors demonstrated that the combinational treatment with mTORC1 inhibition and ferroptosis induction could lead to near‐complete tumor regression in xenograft mouse models with PI3K‐mutated breast cancer or PTEN‐defective prostate cancer.^[^ ^120^ ^]^ As shown by the reports above, ferroptosis could be a powerful tool for tumor therapy in a clinical context.

The distinct biochemical characteristics of ferroptosis also offer emerging opportunities for the development of new combinational therapies, which may afford improved antitumor efficacy compared to mono‐therapies due to the beneficial therapeutic interactions. Typically, it was demonstrated that CD8+ T cells activated by immunotherapy could release IFN*γ* to inhibit the expression of SLC7A11 in tumor cells and impair the cystine uptake, thus inducing tumor cell ferroptosis.^[^ ^128^ ^]^ Extending from the interaction between ferroptosis and immunotherapy, the authors designed an engineered cystine‐degrading enzyme called cyst(e)inase, which could synergize with PD‐L1 immunotherapy for enhanced tumor inhibition efficacy. Meanwhile, some studies have shown that ferroptosis was a contributing factor to the radiotherapy‐mediated tumor inhibition, and treating tumor cells with ferroptosis inducers can effectively promote their sensitivity to radiotherapy via compromising the ionizing radiation‐induced SLC7A11/GPX4 induction and disrupting the ROS‐scavenging system.^[^ ^129^ ^]^ Ferroptosis inducers may also cooperate with other ROS‐reliant chemotherapeutic drugs such as cisplatin by simultaneously elevating the nutrient and oxidative stresses.^[^ ^130^ ^]^ These findings strongly support the therapeutic synergism between ferroptosis and other mainstream antitumor modalities including chemotherapy, radiotherapy, and immunotherapy, potentiating novel combinational therapies with improved clinical transability.

By exploiting the markedly upregulated glucose uptake in hyperglycolytic tumor cells, glucose oxidase (GoX), a natural‐occurring enzyme that has been extensively explored for biocatalytic therapy, demonstrates significant relevance for ferroptosis‐based tumor treatment.^[^ ^131^, ^132^ ^]^ Typically, GoX could convert glucose into non‐metabolizable gluconic acid and H_2_O_2_, of which the latter could act as an endogenous oxidant to overcome the low H_2_O_2_ abundance in tumor cells under bioenergetic stress to enhance lipid peroxidation, thus potentially amplifying the ferroptotic damage.^[^ ^133^, ^134^, ^135^ ^]^ Nevertheless, it was also acknowledged that the GoX usually demonstrates indiscriminate cytotoxicity and is very susceptible to denaturation upon entering the physiological environment, thus necessitating the development of new delivery technologies to perverse its bioactivity and enhance the tumor‐targeted uptake.^[^ ^136^, ^137^ ^]^ Remarkably, recent reports collectively demonstrated that the clinical translation of GoX‐assisted ferroptosis therapy could be substantially accelerated by the integration of nanotechnology.^[^ ^135^, ^138^, ^139^ ^]^ From an overall perspective, nanoparticulate therapeutic systems usually have superior tumor‐targeting effect than free drugs as well as multiplexing capabilities for other non‐therapeutic applications such as imaging or diagnosis. For instance, Yang et al. synthesized cancer cell membrane‐coated metal‐organic framework for the facile incorporation of GoX, Fe^3+^ ions, and doxorubicin for tumor‐targeted triplet ferroptosis/chemotherapy/immunotherapy.^[^ ^138^ ^]^ The released GoX could catalyze the oxidation of glucose to produce abundant H_2_O_2_, while the Fe^3+^ would react with the intracellular GSH and reduced to Fe^2+^, which would not only break down the antioxidative defense in tumor cells but also confer greater reactivity of the Fenton system for ferroptosis initiation. Similarly, GoX has also been used in conjunction with other metal ions with Fenton catalytic activity such as manganese and copper. Typically, Fu et al. developed an intelligent nanocatalytic theranostic system based on polyethylene‐glycol‐modified GoX (PEG‐GoX) to selectively enhance the production of hydroxyl radicals in tumor cells via Fenton‐like reaction routes.^[^ ^140^ ^]^ The authors employed PEG‐GoX as a biomolecular template to obtain doxorubicin‐loaded copper‐incorporated calcium phosphate nanoparticles. The nanoparticles could be rapidly hydrolyzed in tumor cells and release the Cu^2+^ ions, PEG‐GoX, and doxorubicin. The Cu^2+^ ions could react with the glutathione in the tumor intracellular compartment and be reduced to Cu^+^, leading to the restoration of its Fenton‐like catalytic reactivity. Meanwhile, PEG‐GoX could rapidly deprive the tumor cells of glucose and enhance the generation of H_2_O_2_, which could readily supply the Cu^+^‐mediated production of hydroxyl radicals. The combination of GoX and Fenton catalytic systems enhanced the hydroxyl radical production efficiency without inducing apparent toxic side effects in vitro and in vivo, further demonstrating the excellent clinical translatability of this strategy for ferroptosis‐based tumor therapy.

Alternative to the usage of GoX, some synthetic energy metabolism inhibitors have also demonstrated promotional effects on ferroptosis‐based treatment. Typically, Song et al. demonstrated in a recent report that pyruvate dehydrogenase kinase 4 (PDK4) was a top gene that regulated ferroptosis resistance in multiple cancerous cell lines, which could directly inhibit the activity of pyruvate dehydrogenase (PDH) and repress the PDH‐mediated pyruvate oxidation, thus preventing the glucose‐derived pyruvate from entering the TCA cycle and reducing the production of ferroptosis‐associated PUFAs to enhance the ferroptosis resistance.^[^ ^141^ ^]^ Dichloroacetate (DCA), a PDK inhibitor that has already been approved for the treatment of metabolic diseases such as lactic acidosis and diabetes, could readily inhibit the activity of PDK4 in tumor cells and enhance their susceptibility to the ferroptosis induction by imidazole ketone erastin. Nevertheless, considering the universal presence of PDK4 in both normal cells and tumor cells as well as the unsatisfactory pharmacokinetic features of DCA, new nanoformulation strategies are still urgently needed for enhancing the tumor‐targeted delivery of DCA. Alternatively, Guo et al. exploited the elevated PPP activities in tumor cells to develop an electron‐accepting micelle for tumor‐targeted ferroptosis therapy, which comprised of a PEG backbone modified with nitroimidazole‐conjugated polypeptide through a sheddable azobenzene linker.^[^ ^142^ ^]^ As the hypoxic tumor microenvironment favors the expression of nitroreductase and NADPH quinone dehydrogenase 1 (NQO1),^[^ ^143^ ^]^ the azobenzene linkers could be readily cleaved by reacting with the NQO1 and NADPH to remove the PEG moieties and enhance the uptake of nitroimidazole, which could afterward react with nitroreductase and NADPH to further deplete the intracellular NADPH, leading to the impairment of both the GSH and Trx redox cycles for ferroptosis induction. Considering the examples above, It is anticipated that the integration with nanotechnology could profoundly improve the efficacy, safety, and controllability of energy metabolism regulation in tumor cells for ferroptosis treatment.

## 7 Conclusion

The rewiring of energy metabolism in tumor cells is one of their most important metabolic features, which is necessary to provide the energy for sustaining the tumor growth and proliferation. Targeting vital processes in the tumor energy metabolism may offer the opportunity to regulate tumor susceptibility to ferroptosis and potentiate novel and effective ferroptosis‐based tumor therapies. Nevertheless, despite these therapeutic opportunities, the clinical translation of ferroptosis‐based tumor therapies is impeded by a series of scientific and practical issues. For instance, metabolic inhibitors that could specifically and precisely inhibit those tumor‐unique energy metabolic activities have yet to be developed, which should have optimal efficacy and safety profiles under clinical conditions. Meanwhile, the actual effectiveness of tumor ferroptosis sensitization through energy metabolic interference remains to be elucidated. It is also necessary to identify and summarize characteristic features of tumor indications that have positive response to ferroptosis therapies after energy metabolic interference, as well as its potential impact on the tumor immunogenicity. Nevertheless, It is also important to note that the clinical translation of these energy‐metabolism‐targeted ferroptosis‐inducing strategies is still impeded by some practical obstacles. For instance, most of the potential therapeutic targets in the energy metabolism pathways are universally present in both normal cells and cancer cells. Moreover, the pharmacokinetics of existing regulatory agents for tumor energy metabolism is still not satisfactory. These issues highlight the necessity of developing new nanotherapeutic strategies to enhance the antitumor efficacy of these energy metabolism regulators while lowering their potential adverse side effects, which may open up new avenues for improving ferroptosis‐based therapies in a clinical context.

## Conflict of Interest

The authors declare no conflict of interest.
